# Epidemic Profile of Maternal Syphilis in China in 2013

**DOI:** 10.1155/2016/9194805

**Published:** 2016-02-11

**Authors:** Lixia Dou, Xiaoyan Wang, Fang Wang, Qian Wang, Yaping Qiao, Min Su, Xi Jin, Jie Qiu, Li Song, Ailing Wang

**Affiliations:** ^1^National Center for Women and Children's Health, China CDC, No. 12 Dahuisi Road, Haidian District, Beijing 100081, China; ^2^National Health and Family Planning Commission, No. 14 Zhichun Road, Haidian District, Beijing 100088, China

## Abstract

*Objective*. The aim of this study was to investigate the epidemiological characteristics and adverse pregnancy outcomes of pregnant women with syphilis infection in China.* Methods*. Data were from China's Information System of Prevention of Mother-to-Child Transmission of Syphilis Management. Women who were registered in the system and delivered in 2013 were included in the analysis.* Results*. A total of 15884 pregnant women with syphilis infection delivered in China in 2013. 79.1% of infected women attended antenatal care at or before 37 gestational weeks; however, 55.4% received no treatment or initiated the treatment after 37 gestational weeks. 14.0% of women suffered serious adverse pregnancy outcomes including stillbirth/neonatal death, preterm delivery/low birth weight, or congenital syphilis in newborns. High maternal titer (≥1 : 64) and late treatment (>37 gestational weeks)/nontreatment were significantly associated with increased risk of congenital syphilis and the adjusted ORs were 1.88 (95% CI 1.27 to 2.80) and 3.70 (95% CI 2.36 to 5.80), respectively.* Conclusion*. Syphilis affects a great number of pregnant women in China. Large proportions of women are not detected and treated at an early pregnancy stage. Burden of adverse pregnancy outcomes is high among infected women. Comprehensive interventions still need to be strengthened to improve uptake of screening and treatment for maternal syphilis.

## 1. Introduction

Pregnant women with syphilis infection can transmit the infection to their fetus and are at great risk for adverse pregnancy outcomes, such as miscarriage, stillbirth, preterm delivery, neonatal death, or congenital syphilis. Up to 80% of the women without effective treatment will experience these serious adverse pregnancy outcomes [1]. The harm caused by maternal and congenital syphilis among children may exceed that of HIV in developing countries [2]. Fortunately, adverse pregnancy outcomes including congenital syphilis can be effectively prevented by universal syphilis screening at an early pregnancy stage and treatment of those infected with penicillin, which can provide immediate benefits to both the infected mother and her fetus/infants. These interventions have been proved to be cost-effective, inexpensive, and feasible.

China has seen a dramatic resurgence of syphilis epidemic following the substantial changes in the society since mid-1980s [3]. Along with the prevalence of syphilis among adults, congenital syphilis has become a public health problem in China and raised wide concern over its impact on children' health. It is reported that congenital syphilis has increased dramatically from 0.01 cases per 100 000 live births in 1991 to 69.9 cases per 100 000 live births in 2013, according to China's national sexually transmitted disease surveillance system [3, 4].

In recent years, China has endorsed a comprehensive strategy for the control of maternal syphilis and elimination of congenital syphilis. In 2011, China released the National Implementation Guidelines on Integrated Prevention of Mother-to-Child Transmission (iPMTCT) of HIV, syphilis, and HBV Programme. This programme is supported by government fund. According to the guidelines, cross-sectional collaboration and integrated approaches have been applied for maternal syphilis controlling and congenital syphilis elimination. Universal syphilis screening, counselling, and education are provided for all pregnant women, ideally at their first antenatal care visit. Either treponemal test or nontreponemal test will do for initial syphilis screening, though treponemal test is highly recommended. For those with positive syphilis tests appropriate treatment will be initiated promptly with at least 2 courses of penicillin treatment, one at early pregnancy stage and one at the third trimester. For each course women will receive either benzathine or procaine penicillin treatment by intramuscular injection, as recommended by the guidelines. Infants born to mothers with syphilis-infection will be followed up up to 18 months, until diagnosis of congenital syphilis is confirmed or excluded. Meanwhile, infants born to mother with inadequate treatment or no treatment will receive prophylactic benzathine penicillin treatment. Infants with confirmed congenital syphilis will be referred to specific health institutions for further treatment. In addition, the programme has established a maternal syphilis surveillance system, that is, China's Information System of Prevention of Mother-to-Child Transmission of Syphilis Management, in order to continuously monitor disease burden and trends of maternal and congenital syphilis in China.

However, maternal syphilis has been poorly investigated in China. There are only a few studies that reported the epidemic of maternal syphilis in local areas in China. Using nationwide surveillance data this paper aims to describe the epidemiological characteristics of pregnant women with maternal syphilis and their pregnancy outcomes in China in 2013. We also tried to identify the missed opportunities for maternal syphilis control and congenital syphilis prevention. These analyses will help raise the awareness of policy makers, researchers, and other stakeholders on this public health problem and improve intervention implementation on maternal and congenital syphilis control.

## 2. Methods

**Figure 1:**
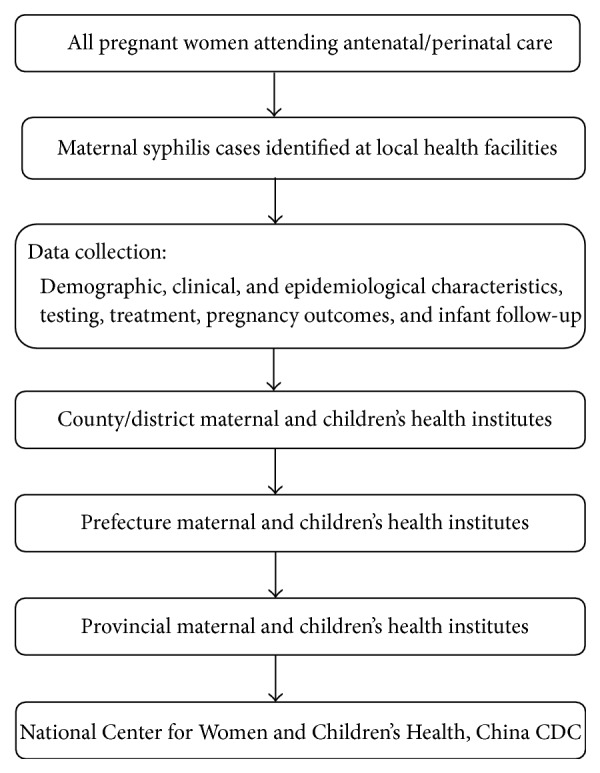
Reporting structure of China's Information System of Prevention of Mother-to-Child Transmission of Syphilis Management.

China's Information System of Prevention of Mother-to-Child Transmission of Syphilis Management is a nationwide, health facility-based case report system. The system was established in 2011 and has been used to monitor and evaluating the prevalence of maternal syphilis and congenital syphilis in China. Surveillance of maternal syphilis is conducted through mandatory case-reporting by health facilities including general hospitals, maternal and children's hospitals, and other health providers across China, involving all 31 provinces, municipalities, and autonomous regions in the country. Diagnosis of maternal syphilis is based on at least one of the following situations in pregnant women: (1) positive treponemal and nontreponemal tests; (2) laboratory confirmation of* Treponema pallidum* in clinical specimens by dark-field microscopy; or (3) reactive treponemal antibody test of IgM. Pregnant women who met the diagnostic criteria are required to be registered in the system and followed up through pregnancy and postpartum period. Maternal syphilis case-reporting moved from paper reports to electronic online submission from 2013.  Figure 1 shows the reporting structure of the surveillance system.

The surveillance system collected maternal syphilis data using standard forms. Relevant information on maternal syphilis was extracted by health staff from medical records. For each maternal syphilis case, data collected include information on demographic characteristics (e.g., maternal age, ethnicity, marital status, occupation, and education), maternal syphilis information (e.g., diagnostic evidence, stage of syphilis, route of acquisition, sexual partner's syphilis status, and laboratory testing results), and pregnancy outcomes and infant follow-up information (e.g., mode of delivery, preterm delivery, low birth weight, and congenital syphilis).

We used data extracted from the above surveillance system in this study. Pregnant women who met the following criteria and their children were included in the analysis: (1) syphilis screening tests positive during pregnancy and delivery at health facilities; (2) being diagnosed with syphilis; (3) being registered in the surveillance system; (4) delivered at gestational age of 28 weeks or more in 2013. Newborns with congenital syphilis born to women who received no syphilis tests were excluded from the analyses.

Migrating people were defined as those who lived in a different city/prefecture other than the place where their household was registered. The definition of syphilis stage is in accordance with the national guidelines [5]. In brief, primary syphilis is a stage of* Treponema pallidum* infection characterized by one or more chancres and demonstration of* T. pallidum* in clinical specimens by dark-field microscopy or reactive nontreponemal and treponemal tests. Secondary syphilis is characterized by maculopapular rash and often with lymphadenopathy and laboratory criteria as for primary syphilis. Tertiary syphilis is defined as a case with a history of primary, secondary, or latent syphilis that has clinical cardiovascular or central nervous system symptoms and signs and reactive nontreponemal tests or elevated cerebrospinal fluid protein or leukocyte count. Latent syphilis is defined as an asymptomatic case that has reactive nontreponemal and treponemal tests. Inadequate treatment for maternal syphilis was defined as nontreatment or nonpenicillin treatment, less than 2 completed courses of treatment during pregnancy, or less than 2 weeks between the 2 courses of treatment. Preterm delivery was defined as a delivery that occurs before 37 gestational weeks. Low birth weight (LBW) was defined as birth weight of a live-born baby of less than 2,500 g at birth. Stillbirth was defined as death of a fetus of at least 28 gestational weeks. Neonatal death was defined as death of live-born baby who died during the first week after birth. Congenital syphilis in newborn was defined as having at least one of the following situations in infant who was younger than 6 weeks and born to syphilis infected mother: (1) positive treponemal test and high titer of nontreponemal test (a value 4-fold higher than that of his/her mother's before delivery); (2) laboratory confirmation of* Treponema pallidum* in clinical specimens by dark-field microscopy; or (3) reactive treponemal antibody test of IgM. “Any adverse pregnancy outcomes” are a composite outcome in our analysis, which include preterm delivery, LBW, stillbirth, neonatal death, and congenital syphilis in newborns.

Statistical analysis was performed using SPSS 20.0. Values for categorical variables were presented as numbers and percentages. Values for continuous variables were presented as means and standard deviations (SD). Comparisons were made between regions of eastern, central, and western China [6] using *χ* ^2^ statistic for categorical variables and *F* statistic (analysis of variances) for continuous variables. *P* values < 0.05 were considered of statistical significance. Crude ORs (cOR) and adjusted ORs (aOR) were calculated using logistic regression to explore potential factors associated with congenital syphilis.

## 3. Results

### 3.1. Overall Characteristics of the Population

In 2013, a total of 15884 pregnant women with laboratory-confirmed syphilis infection delivered and were registered in Information System of Prevention of Mother-to-Child Transmission of Syphilis Management in China. Proportions of infected women from eastern, central, and western China were 41.7%, 23.9%, and 34.4%, respectively.  Table 1 shows the demographic characteristics of these infected pregnant women. The majority (79.5%) of the women were aged between 20 and 34 years old. In eastern and central China, women of minority ethnicity accounted for less than 10%, while in the western area nearly 40% were minority. 23.4% of infected women were migrating people. 6% were unmarried (including those being single, cohabitated, divorced, or widowed) during the index pregnancy. 41.1% were housewives or unemployed, ranging from 25.9% in western area to 52.5% in eastern area. 79.1% of infected women attended antenatal care at or before 37 gestational weeks, while 16.8% attended antenatal care after 37 gestational weeks and 4.1% were unknown.

### 3.2. Clinical Characteristics of Pregnant Women with Syphilis Infection

Table 2 shows the syphilis characteristics of infected pregnant women in China in 2013. 18.3% of women reported that they had had syphilis infection before the index pregnancy. Proportions of women with latent syphilis, primary syphilis, secondary syphilis, and tertiary syphilis were 67.6%, 6.2%, 1.0%, and 0.5%, respectively, leaving 24.7% of those with syphilis stage being unknown. Only 55.6% of infected women had their syphilis detected during pregnancy, and more than 40% remained undetected until delivery or postpartum period. Proportions of women who initiated the treatment for maternal syphilis during the first and second trimesters were 12.4% and 19.8%, respectively. 55.4% were untreated or initiated the treatment after 37 gestational weeks. Nearly 60% of women received penicillin treatment for maternal syphilis, while 37.9% received no treatment at all. 68.8% of infected women's husband/sexual partners did not undertake any syphilis tests and their infection status was unknown during the entire period of the index pregnancy. Among those who received syphilis tests, 26.3% were reported to test positive for syphilis.

### 3.3. Pregnancy Outcomes and Complications of Pregnant Women with Syphilis Infection

Table 3 shows the pregnancy outcomes of pregnancy women with syphilis infection in China in 2013. The mean gestational age at delivery was 39.2 weeks. 51.9% of women gave birth by vaginal delivery and 46.3% by caesarean section. 14.0% of women suffered adverse pregnancy outcomes, and proportions of women who experienced stillbirth/neonatal death, preterm delivery/low birth weight, and neonatal congenital syphilis were 2.9%, 10.5%, and 3.0%, respectively.

### 3.4. Associations between Clinical Characteristics of Syphilis Infected Pregnant Women and Congenital Syphilis in Newborns

Table 4 shows that in both univariable and multivariable models, maternal titer of nontreponema and gestational week for the first treatment were statistically associated with congenital syphilis in newborns. Women with high maternal titer (≥1 : 64) had greater risk of having babies with congenital syphilis than those with low titer (≤1 : 4) (adjusted OR (aOR) 1.88, 95% CI 1.27 to 2.80). Compared with early treatment for infection (≤14 gestational weeks), treatment initiated during 29–37 weeks and late treatment (>37 gestational weeks)/nontreatment were significantly associated with increased risk of congenital syphilis (aOR 3.20, 95% CI 1.94 to 5.27, and aOR 3.70, 95% CI 2.36 to 5.80, resp.).

## 4. Discussion

Data in this study came from China's Information System of Prevention of Mother-to-Child Transmission of Syphilis Management. Identified maternal syphilis cases are mandatory to be registered to the system in China. The established system can provide useful evidence for policy-makers and other stakeholders on planning, implementing, and evaluating public health policies on maternal and congenital syphilis control. In our study, only pregnant women with syphilis infection that delivered in 2013 were eligible for inclusion. Hence, a total of 15884 women were included in the analysis. We analyzed the demographic, clinical, and syphilis related characteristics of these infected pregnant women in China, as well as their pregnancy outcomes. Our findings indicate that most pregnant women with syphilis infection received penicillin treatment; however, there were still large proportions of women that had not been detected and treated at an early pregnancy stage. Vast intervention opportunities were missed to prevent adverse pregnancy outcomes caused by maternal syphilis.

China commenced the integrated programme of prevention of mother-to-child transmission (iPMTCT) of human immunodeficiency virus (HIV), syphilis, and hepatitis B virus (HBV) in 2010. Free HIV, syphilis, and HBV tests are provided to all pregnant women. In 2013, the syphilis testing coverage in pregnant women was 96.40% [7]. All syphilis infected women will be provided free intramuscular penicillin treatment.

Migrating women, women with lower education level, and women who are jobless or housewives/farmers are usually socioeconomically disadvantaged. In our study, we found that this less advantaged population accounted for a large proportion of maternal syphilis cases in China. Former studies showed that prevalence of maternal syphilis in migrating population was higher than that in local residents and their compliance to treatment tended to be poor [8]. Extra attention and commitment should be made to promote the uptake of syphilis tests and compliance of treatment in this vulnerable population.

China's iPMTCT guidelines recommend that all pregnant women should be subjected to a syphilis screening either with nontreponemal tests or treponemal tests at their first antenatal care visit. Early prenatal care is an important component for prevention of congenital syphilis because it can facilitate early detection of maternal syphilis and prompt treatment for those with positive tests [9, 10]. It was estimated that one week's delay in initiation of antenatal care could approximately raise the risk of congenital syphilis by 10% [11]. Our study showed that delayed or absence of antenatal care was not uncommon among women with syphilis infection. Only 40% initiated antenatal care during the first trimester. Around 56% of women with maternal syphilis were identified during pregnancy and more than 40% were diagnosed at delivery or postpartum period. Several reasons are probably behind this worrying situation of low recognition of the disease. First, screening tests for syphilis might be unavailable temporally in some clinics because test kits were out of stock; second, health providers might have not asked women to undertake the tests; third, but not the least, women with syphilis infection might be reluctant to take the tests either because of worries about stigma, costs, or beliefs of unnecessary. All sexual partners of pregnant women should undertake syphilis tests and those with positive test should receive treatment, as recommended by China's iPMTCT guidelines. We found that syphilis tests were performed for about 30% of sexual partners of pregnant women with syphilis infection. For nearly 70% of the partners the syphilis infection status was unknown. This could put women at high risk of reinfection of the disease, even after effective treatment. To address these issues, testing and counselling services should be further strengthened at antenatal clinics.

Penicillin treatment is necessary to improve health outcomes for both women and children [12, 13]. China's iPMTCT guidelines recommend that all pregnant women with syphilis infection should receive adequate treatment to prevent congenital syphilis. In 2013, less than a third of women with syphilis infection initiated treatment before the third trimester, while more than a third received no treatment at all. Hence, a large proportion of pregnant women were untreated or inadequately treated, which posed great risk for congenital syphilis. Patient education, along with free treatment services, should be improved to increase women's awareness of the infection and their willingness for treatment. In addition, undocumented treatment needs to be investigated to get better understanding of the treatment situation. Our study showed that 14% of women suffered serious adverse pregnancy outcomes, including stillbirth/neonatal death (10.5%), preterm delivery/low birth weight (2.9%), or neonatal congenital syphilis (3.0%). Previous studies in China showed that in general population the prevalence rates of stillbirth and preterm delivery were around 0.3% [14] and 3.8% [15], respectively, which is much lower than those with syphilis infection. Infected women were still at great risk for adverse pregnancy outcomes and maternal syphilis continues to be an important cause for morbidity and mortality in pregnancy. However, adverse pregnancy outcomes caused by maternal syphilis can be almost entirely averted by early detection and prompt treatment [16]. The high prevalence of adverse pregnancy outcomes highlighted the gaps existing in current antenatal cares.

In 2007, World Health Organization (WHO) called on global elimination of congenital syphilis by 2015 and developed strategies to combat the disease, including strong political will, improved access to antenatal care, screening and treatment, and surveillance system [17]. In response, China has shown great political will to achieve this goal by introducing universal maternal syphilis screening and treatment for positive cases, along with other interventions, such as improving access, uptake, and quality of early prenatal care, providing education and counseling, and sexual partner testing and treatment if necessary. Currently, high antenatal care attendance level, cost-effective diagnostic testing, and the widespread availability of penicillin are already in place across the country. But maternal and congenital syphilis continue being a serious public health problem and elimination of congenital syphilis is still a big challenge to China. Barriers that hamper the uptake of syphilis testing and treatment during pregnancy still exist and need to be identified and tackled. More commitment should be taken to enhance the implementation of maternal and congenital syphilis controlling programme.

This study has several limitations. First, our data were from China's Information System of Prevention of Mother-to-Child Transmission of Syphilis Management, which is a passive surveillance system. It might be biased by low incidence reporting due to underdetection, misclassification, and underreporting, since data collection on maternal syphilis relies on health facility reporting. Second, only women who delivered at a gestational age of no less than 28 weeks were included in the analysis. Hence, we might underestimate the situation of maternal syphilis in China, for many may have miscarriages and fetal loss in the early stage of pregnancy. Third, only neonatal congenital syphilis cases rather than all congenital syphilis cases were included in our study. Because many infected infants are asymptomatic at birth or with subtle and nonspecific symptoms and maternal nontreponemal and treponemal antibodies can cross the placenta, this makes the diagnosis of congenital syphilis problematic. So infants born to mother with syphilis infection need to be evaluated with series of serological tests and followed up up to 18 months before diagnosis of congenital syphilis can be made or be excluded. However, by the time of our analysis, these data on all congenital syphilis cases were not available and were not analyzed in this paper.

According to our knowledge, this is the first study regarding the country's epidemic profile of maternal syphilis in China. Through our analysis on antenatal screening, maternal syphilis treatment, and pregnancy outcomes using the national data, we can see that the maternal syphilis picture in China is alarming. Integrated interventions on maternal syphilis still need to be further strengthened to promote maternal and children's health. Government should commit more to investment in infrastructure of public health, health provider capacity building, and population education. In addition, more studies are needed to explore barriers and strategies to elimination of congenital syphilis in China.

## 5. Conclusions

Syphilis continues to affect a large number of pregnant women in China. Universal maternal syphilis screening and treatment for positive cases have been in place for several years across the country, yet there were still large proportions of women that had not been detected and treated at an early pregnancy stage. Burden of adverse pregnancy outcomes is high among infected women. Barriers that hamper the uptake of syphilis testing and treatment during pregnancy still exist and need to be identified and tackled. China's integrated strategies for maternal syphilis control need to be further strengthened to improve access to antenatal care, syphilis screening, and treatment during early pregnancy.

## Figures and Tables

**Table 1:** Demographic characteristics of pregnant women with syphilis infection in China in 2013, by region^a^.

Characteristic	Total	Region			*P* value
		Eastern	Central	Western	
Maternal age (years)					
<20	1138 (7.2%)	437 (6.6%)	221 (5.8%)	480 (8.8%)	<0.001
20–34	12627 (79.5%)	5310 (80.2%)	3072 (80.9%)	4245 (77.6%)	
≥35	2119 (13.3%)	872 (13.2%)	505 (13.3%)	742 (13.6%)	
Ethnicity					
Han	13100 (82.5%)	6229 (94.1%)	3558 (93.7%)	3313 (60.6%)	<0.001
Minority	2784 (17.5%)	390 (5.9%)	240 (6.3%)	2154 (39.4%)	
Residency status					
Local resident	12170 (76.6%)	4233 (64.0%)	3304 (87.0%)	4633 (84.7%)	<0.001
Migrating people	3714 (23.4%)	2386 (36.0%)	494 (13.0%)	834 (15.3%)	
Marital status					
Single	643 (4.0%)	406 (6.1%)	95 (2.5%)	142 (2.6%)	<0.001
First married	13658 (86.0%)	5662 (85.5%)	3339 (87.9%)	4657 (85.2%)	
Remarried	1267 (8.0%)	397 (6.0%)	302 (8.0%)	568 (10.4%)	
Cohabitated	235 (1.5%)	105 (1.6%)	47 (1.2%)	83 (1.5%)	
Divorced/widowed	81 (0.5%)	49 (0.7%)	15 (0.4%)	17 (0.3%)	
Education					
Primary or lower	2982 (18.8%)	955 (14.4%)	469 (12.3%)	1558 (28.5%)	<0.001
Junior middle school	8290 (52.2%)	3313 (50.1%)	2166 (57.0%)	2811 (51.4%)	
Senior middle school	2921 (18.4%)	1441 (21.8%)	743 (19.6%)	737 (13.5%)	
College or above	896 (5.6%)	502 (7.6%)	191 (5.0%)	203 (3.7%)	
Unknown	795 (5.0%)	408 (6.2%)	229 (6.0%)	158 (2.9%)	
Occupation					
Farmers	5828 (36.7%)	1315 (19.9%)	1294 (34.1%)	3219 (58.9%)	<0.001
Housewife/unemployed	6521 (41.1%)	3477 (52.5%)	1630 (42.9%)	1414 (25.9%)	
Others	3535 (22.3%)	1827 (27.6%)	874 (23.0%)	834 (15.3%)	
Gravidity					
1	4065 (25.6%)	1571 (23.7%)	1200 (31.6%)	1294 (23.7%)	<0.001
2-3	8637 (54.4%)	3656 (55.2%)	1953 (51.4%)	3028 (55.4%)	
>3	3182 (20.0%)	1392 (21.0%)	645 (17.0%)	1145 (20.9%)	
Parity					
0	5289 (33.3%)	2302 (34.8%)	1331 (35.0%)	1656 (30.3%)	<0.001
1-2	9778 (61.6%)	4013 (60.6%)	2331 (61.4%)	3434 (62.8%)	
>3	817 (5.1%)	304 (4.6%)	136 (3.6%)	377 (6.9%)	
First antenatal care (gestational weeks)					
≤14	6379 (40.2%)	2556 (38.6%)	1687 (44.4%)	2136 (39.1%)	<0.001
15–28	4175 (26.3%)	2003 (30.3%)	824 (21.7%)	1348 (24.7%)	
29–37	2007 (12.6%)	849 (12.8%)	431 (11.3%)	727 (13.3%)	
>37	2665 (16.8%)	861 (13.0%)	718 (18.9%)	1086 (19.9%)	
Unknown	658 (4.1%)	350 (5.3%)	138 (3.6%)	170 (3.1%)	

^a^Values are given as number (percentage) unless otherwise indicated.

**Table 2:** Clinical characteristics of pregnant women with syphilis infection in China in 2013, by region^a^.

Characteristic	Total	Region			*P* value
		Eastern	Central	Western	
History of syphilis infection					
Yes	2910 (18.3%)	1537 (23.2%)	566 (14.9%)	807 (14.8%)	<0.001
No	12974 (81.7%)	5082 (76.8%)	3232 (85.1%)	4660 (85.2%)	
Maternal syphilis stage					
Latent syphilis	10742 (67.6%)	4751 (71.8%)	2193 (57.7%)	3798 (69.5%)	<0.001
Primary syphilis	981 (6.2%)	421 (6.4%)	234 (6.2%)	326 (6.0%)	
Secondary syphilis	158 (1.0%)	81 (1.2%)	48 (1.3%)	29 (0.5%)	
Tertiary syphilis	73 (0.5%)	39 (0.6%)	17 (0.4%)	17 (0.3%)	
Unknown	3930 (24.7%)	1327 (20.0%)	1306 (34.4%)	1297 (23.7%)	
Period of maternal syphilis detection					
During pregnancy	8829 (55.6%)	4136 (62.5%)	1728 (45.5%)	2965 (54.2%)	<0.001
At delivery	5246 (33.0%)	1675 (25.3%)	1619 (42.6%)	1952 (35.7%)	
Postpartum	1722 (10.8%)	771 (11.6%)	430 (11.3%)	521 (9.5%)	
Other	87 (0.5%)	37 (0.6%)	21 (0.6%)	29 (0.5%)	
Maternal titer of nontreponema					
≤1 : 4	11118 (70.0%)	4780 (72.2%)	2587 (68.1%)	3751 (68.6%)	<0.001
1 : 8–1 : 32	3736 (23.5%)	1405 (21.2%)	1006 (26.5%)	1325 (24.2%)	
≥1 : 64	565 (3.6%)	261 (3.9%)	111 (2.9%)	193 (3.5%)	
Unknown	465 (2.9%)	173 (2.6%)	94 (2.5%)	198 (3.6%)	
Gestational week for the first treatment					
≤14	1972 (12.4%)	1033 (15.6%)	297 (7.8%)	642 (11.7%)	<0.001
15–28	3151 (19.8%)	1498 (22.6%)	500 (13.2%)	1153 (21.1%)	
29–37	1966 (12.4%)	815 (12.3%)	388 (10.2%)	763 (14.0%)	
>37 or untreated	8795 (55.4%)	3273 (49.4%)	2613 (68.8%)	2909 (53.2%)	
Drug of treatment					
Penicillin	9386 (59.1%)	3940 (59.5%)	1785 (47.0%)	3661 (67.0%)	<0.001
Nonpenicillin	485 (3.1%)	253 (3.8%)	99 (2.6%)	133 (2.4%)	
Untreated	6013 (37.9%)	2426 (36.7%)	1914 (50.4%)	1673 (30.6%)	
Syphilis testing of husband/sexual partner					
Negative	3654 (23.0%)	1616 (24.4%)	890 (23.4%)	1148 (21.0%)	<0.001
Positive	1302 (8.2%)	542 (8.2%)	275 (7.2%)	485 (8.9%)	
Untested/unknown	10928 (68.8%)	4461 (67.4%)	2633 (69.3%)	3834 (70.1%)	

^a^Values are given as number (percentage) unless otherwise indicated.

**Table 3:** Pregnancy outcomes of women with syphilis infection in China in 2013, by region^a^.

Characteristic	Total	Region			*P* value
		Eastern	Central	Western	
Gestational weeks at delivery (mean ± SD)	39.2 ± 2.2	39.1 ± 2.3	39.2 ± 2.3	39.3 ± 2.1	<0.001
Mode of delivery					
Spontaneous VD^b^	7900 (49.7%)	3388 (51.2%)	1473 (38.8%)	3039 (55.6%)	<0.001
Instrumental VD^b^	351 (2.2%)	107 (1.6%)	102 (2.7%)	142 (2.6%)	
Elective CS^c^	4355 (27.4%)	1821 (27.5%)	1390 (36.6%)	1144 (20.9%)	
Emergency CS^c^	3005 (18.9%)	1055 (15.9%)	820 (21.6%)	1130 (20.7%)	
Unknown	273 (1.7%)	248 (3.7%)	13 (0.3%)	12 (0.2%)	
Stillbirth/neonatal death					
Yes	454 (2.9%)	200 (3.0%)	121 (3.2%)	133 (2.4%)	0.059
No	15430 (97.1%)	6419 (97.0%)	3677 (96.8%)	5334 (97.6%)	
Preterm delivery/low birth weight					
Yes	1665 (10.5%)	728 (11.0%)	408 (10.7%)	529 (9.7%)	0.051
No	14219 (89.5%)	5891 (89.0%)	3390 (89.3%)	4938 (90.3%)	
Neonatal congenital syphilis					
Yes	478 (3.0%)	191 (2.9%)	181 (4.8%)	106 (1.9%)	<0.001
No	15406 (97.0%)	6428 (97.1%)	3617 (95.2%)	5361 (98.1%)	
Any adverse pregnancy outcomes					
Yes	2223 (14.0%)	946 (14.3%)	598 (15.7%)	679 (12.4%)	<0.001
No	13661 (86.0%)	5673 (85.7%)	3200 (84.3%)	4788 (87.6%)	

^a^Values are given as number (percentage) or mean ± SD unless otherwise indicated; ^b^VD refers to vaginal delivery; ^c^SD refers to caesarean section.

**Table 4:** Logistic regression analysis of syphilis infected pregnant women's clinical characteristics and congenital syphilis in newborns.

	*n* (%)	Univariable		Multivariable^a^	
		cOR^b^ (95% CI)	*P* Value	aOR^c^ (95% CI)	*P* Value
History of syphilis infection					
Yes	74 (2.5%)	—		—	
No	404 (3.1%)	1.23 (0.96, 1.58)	0.104	0.98 (0.76, 1.27)	0.870
Maternal syphilis stage					
Latent syphilis	275 (2.6%)	—		—	
Primary syphilis	38 (3.9%)	1.53 (1.09, 2.17)	0.015	1.38 (0.97, 1.96)	0.071
Secondary syphilis	8 (5.1%)	2.03 (0.99, 4.18)	0.054	1. 58 (0.76, 3.27)	0.220
Tertiary syphilis	0 (0.0%)	NA		NA	
Unknown	157 (4.0%)	1.58 (1.30, 1.93)	<0.001	1.42 (1.15, 1.74)	0.001
Maternal titer of nontreponema					
≤1 : 4	279 (2.5%)	—		—	
1 : 8–1 : 32	150 (4.0%)	1.63 (1.33, 1.99)	<0.001	1.60 (1.30, 1.97)	<0.001
≥1 : 64	29 (5.1%)	2.10 (1.42, 3.11)	<0.001	1.90 (1.28, 2.83)	0.002
Unknown	20 (4.3%)	1.75 (1.10, 2.78)	0.018	1.37 (0.86, 2.19)	0.189
Gestational week for the first treatment					
≤14	21 (1.1%)	—		—	
15–28	49 (1.6%)	1.47 (0.88, 2.45)	0.144	1.41 (0.84, 2.37)	0.192
29–37	67 (3.4%)	3.28 (2.00, 5.37)	<0.001	3.20 (1.94, 5.27)	<0.001
≥37 or untreated	341 (3.9%)	3.75 (2.41, 5.84)	<0.001	3.70 (2.36, 5.80)	<0.001

^a^The model was adjusted for maternal age, residence location, education, and job.

^b^cOR refers to crude odds ratio.

^c^aOR refers to adjusted odds ratio.
